# Guaiacol Vapour Baths in Bronchiectasis

**Published:** 1899-07-29

**Authors:** 


### GUAIACOL VAPOUR BATHS IN BRONCHIECTASIS.

Dr. Hearn Parry,[1] of the Ventnor Hospital for Consumption, records a case of bronchiectasis, with profuse and very fetid expectoration, in which after various other methods, including vapour baths of creasote, had failed, complete relief was obtained by five weeks' treatment by guaiacol vapour baths. The duration of the bath was gradually increased from 10 to 60 minutes. The cavity was situated in the left infra-scapular region, and, with the exception of slight dulness on percussion over the left apex anteriorly, no abnormal physical signs were detected elsewhere. No tubercle bacilli were discovered in the sputa, but the evening temperature was raised, and the patient was evidently suffering from septic poisoning when the treatment by guaiacol was commenced.

[1] Lancet, July 22.

